# The Harrison Antinarcotic Bill Which Takes Effect March 1

**Published:** 1915-02

**Authors:** 


### THE HARRISON ANTINARCOTIC BILL WHICH TAKES EFFECT MARCH 1.

### A DIGEST AND EXPLANATION.

As we stated in these pages last month, the Harrison Antinarcotic Bill (H. R. 6282) was finally passed by Congress, and receive the approval of President Wilson upon December 17, 1914. As provided, this bill will go into effect on March 1, 1915. In order that readers of *Clinical Medicine* may be thoroughly familiar with the provisions of the Act and know exactly what they must do (and what they can not do) to comply with it, we submit herewith a brief digest and explanation. The information presented is based to some extent upon regulations now being drafted by the officials of the Treasury Department. It should be understood, however, that these regulations have not been definitely determined upon and are subject to revision. Should there be any changes affecting the accuracy of this explanation, they will be presented to our readers next month.

*Who are affected by the law.*—"Every person who produces, imports, manufactures, compounds, deals in, dispenses, sells, distributes or gives away opium or coca leaves or any compound, manufacture, salt, derivative, or preparation thereof." This includes every physician, dentist, veterinarian and pharmacist, all of whom are specifically mentioned in the law.

*With what narcotic drugs is this law concerned.*—As stated above, this law affects the traffic in opium or coca leaves and "any compound, manufacture, salt, derivative or preparation thereof." However, there are certain exemptions. Thus, decocainized coca leaves are exempt from the operation of the law; also (see Section 6), it is provided that "This Act shall not be construed to apply to the sale, distribution, giving away, dispensing, or possession of preparations and remedies which do not contain more than two grains of opium, or more than one-fourth of a grain of morphine, or more than one-eighth of a grain of heroin, or more than one grain of codeine, or any salt or derivative of any of them in one fluid ounce, or, if a solid or semisolid preparation, in one avoirdupois ounce; or to liniments, ointments, or other preparations which are prepared for external use only, except liniments, ointments, and other preparations which contain cocaine or any of its salts or alpha or beta eucaine or any of their salts or any synthetic substitute for them: *Provided*, That such remedies and preparations are sold, distributed, given away, dispensed, or possessed as medicines and not for the purpose of evading the intentions and provisions of this Act."

The effect of this exemption is to permit the sale by unregistered dealers of many socalled "patent" and "proprietary" medicines containing small quantities of opium and its derivatives. It also exempts at least one official preparation, i. e., camphorated tincture of opium (i. e., paregoric). This clause is of course a concession to the proprietary and retail drug interests. So far as we can discover, every tablet, pill, and granule, and most of the liquid preparations containing opium or its alkaloids, fall under the operations of the law, and every physician dispensing or prescribing them will be compelled to take out a license.

*The license and how it is secured.*—Every physician, dentist, veterinarian and pharmacist, whether he dispenses or prescribes, must take out a license from the National Government, for which he will pay $1.00. This license must be secured from the Collector of Internal Revenue in the district in which the applicant lives or does business. To secure this license, the applicant must fill out and submit to the Collector an application blank which said Collector will supply. In this blank the physician must state his name, location, and the character of his business or profession.

When the license is issued, the applicant will be given a registry number. It is expected that this number will be a permanent one, and that when the license is issued, from year to year, the same number will go with it. Thus, John Jones, M.D., once registered as No. 456, will remain No. 456 throughout his life. He will be permanently identified with this registry number.

*First Payment for license.*—Inasmuch as the fiscal year of the Internal Revenue Bureau begins on July 1, it is presumed that the first payment for license will cover the period from March 1 to July 1. This would make the first expense for license approximately 34 cents; in that event, the license would have to be renewed upon July 1.

*Order blanks, and routine to be followed in ordering drugs.*—The law provides "That it shall be unlawful for any person to sell, barter, exchange, or give away any of the aforesaid drugs except in pursuance of a written order of the person to whom such article is sold, bartered, exchanged, or given, on a form to be issued in blank for that purpose by the Commissioner of Internal Revenue. Every person who shall accept any such order, and in pursuance thereof shall sell, barter, exchange, or give away any of the aforesaid drugs, shall preserve such order for a period of two years in such a way as to be readily accessible to inspection by any officer, agent, or employe of the Treasury Department duly authorized for that purpose, and the

State, Territorial, District, Municipal, and insular officials named in section five of this Act. Every person who shall give an order as herein provided to any other person for any of the aforesaid drugs shall, at or before the time of giving such order, make or cause to be made a duplicate thereof on a form to be issued in blank for that purpose by the Commissioner of Internal Revenue, and in case of the acceptance of such order, shall preserve such duplicate for said period of two years in such a way as to be readily accessible to inspection by the officers, agents, employes, and officials hereinbefore mentioned.'' (Section 2.)

This means that every physician, dentist, veterinarian, pharmacist, or other dealer ordering from any person whatsoever, any of the narcotic drugs mentioned in this Act, must use in ordering them an official order blank, to be furnished by the Commissioner of Internal Revenue. These blanks will be furnished to the applicant by the local Collector of Internal Revenue. In order to secure them the doctor must make out a requisition blank which this official will supply.

The order blanks themselves must be purchased by the applicant (physician, dentist, veterinarian or pharmacist) and they will cost $1.00 a hundred. They will be made in duplicate, the price being for the 100 originals, duplicate being on the same sheet, probably as a ''stub.'' The law requires that *the buyer* must keep the duplicate blank on file in his office subject to official inspection for a term of two years; while *the seller* must keep the original blank on file subject to inspection for a similar period. It is expected that these order blanks will be stamped with the registry number of the purchaser.

*How does this Act affect the dispensing of narcotic drugs.*—The law specifically provides that ''nothing in this Act shall apply (a) to the dispensing and distribution of any of the aforesaid drugs to a patient by a physician, dentist or veterinary surgeon registered under this Act in the course of his professional practice only.'' There is, however, one restriction, as follows: "The physician, dentist or veterinary surgeon must keep a record of all such drugs dispensed or distributed, showing the amount dispensed or distributed, the date, and the name and address of the patient to whom such drugs are dispensed or distributed," *provided* he does not "personally attend" the patient for whom the drugs dispensed or distributed are designed. These records must be kept, subject to inspection, for a period of two years. There is no restriction on the possession and administration of narcotic drugs by the patient's nurse under the physician's direction.

This means that if a patient is actually under the personal care of the physician or veterinarian, the practitioner is not required to keep a record of the narcotic drugs used. On the other hand, if the patient is not under his direct personal care, that is, if the drugs are sent to this patient by mail or messenger after written, telephonic or telegraphic request, without prior actual personal attendance on the part of the physician, then such records must be kept; in other words, every physician doing a mail-order business, or trying to treat patients without actually seeing them, will be required to keep the full records required of all other dealers.

*How the bill affects the prescribing of narcotic drugs.*—It is expected that each physician prescribing any of the drugs mentioned in this Act will be required to sign his name in full to the prescription, and also to write thereon his registry number and office location. It is also expected that the pharmacist will be required to use reasonable precautions to verify the identity of the persons named on the prescription, and to prevent the drugs specified falling into the hands of improper persons. Such prescriptions must be kept (it is expected) in a separate file, for a period of two years. Except on prescriptions written by registered physicians, dentists and veterinary surgeons, the pharmacist is not allowed to dispense any narcotics whatever, except those specifically exempted by the Act.

(See paragraph: *"With what narcotic drugs is this law concerned."*)

*Inventories of narcotic drugs.*—It is presumed that the Government will require of every manufacturer, dealer and dispenser of these drugs, an inventory of the quantities of narcotics on hand at the time this law goes into operation. However, just what will be required in this connection, we are yet unable to state. We have understood that these inventories must be made by March 5; but full information will doubtless be given by the Government later. Physicians can make no mistake in putting in their stocks at once.

*Penalties.*—The penalties provided for the violation of this Act are very high. It is provided "That any person who violates or fails to comply with any of the requirements of this Act shall, on conviction, be fined not more than $2,000, or be imprisoned not more than five years, or both, in the discretion of the court."

This digest does not attempt to cover the Act in full, but only so much of it as may be of special importance to physicians at the present time. The requirements relative to importers, manufacturers and dealers are extremely rigid.

It will be apparent from the preceding that it will be impossible, or at least dangerous, for any physician, dentist or veterinarian to dispense or prescribe any of the narcotic drugs mentioned in this law after March 1, unless he has obtained a license and secured his registry number and order blanks. We therefore advise every physician to file with his local Collector of Internal Revenue, without delay, his application for license and his request for blanks. When convenient, such applications may be made personally, but if not convenient, they may be made by mail.—*Clinical Medicine.*

